# Latest Approved Therapies for Metastatic Melanoma: What Comes Next?

**DOI:** 10.1155/2013/735282

**Published:** 2013-02-24

**Authors:** Farid Menaa

**Affiliations:** Department of Oncology, Stem Cells and Nanomedicine, Fluorotronics, Inc., 2453 Cades Way, Building C, San Diego, CA 92081, USA

## Abstract

Nowadays, oncogene-directed therapy and immunotherapy represent the two most promising avenues for patients with metastatic melanoma. The recent oncogene-directed therapeutic, vemurafenib, usually produces high level of tumor shrinkage and survival benefits in many patients with *B-RAF* ^V600E^ mutant melanomas, although the fast and high degrees of responses are likely short-lived. Conversely, the newly-approved immunotherapeutic, ipilimumab, produces durable responses in patients presenting CTLA-4 T-cell surface protein. Nevertheless, the possible synergy in combining these two therapeutic strategies primarily rely on the rational design of medical protocols (e.g., sequence and timing of agent administration; drug selectivity; compatibility of combined therapies i.e., adoptive T cell or agents, i.e., MEK inhibitor trametinib, PD-1 and PDL-1 blockers). Improved therapeutic protocols shall overcome therapeutic limitations such as the (i) tolerability and safety (i.e., minimal toxic side-effects); (ii) progression free survival (e.g., reduced relapse disease frequency); (iii) duration response (i.e., decreased drug resistance). Eventually, multidisciplinary approaches are still requested (e.g., genomics for personalized medicine, nanomedicine to overcome low free-drug bioavailability and targeting, systematic search of “melanoma stem cells” to enhance the prognosis and develop more valuable theranostics). In this paper, I will mainly present and discuss the latest and promising treatments for advanced cutaneous melanomas.

## 1. Introduction

Melanoma (from Greek—melas: “dark”) is a tumor originated from malignant transformation of melanocytes (i.e., melanin pigment-producing cells) that can be found in the skin, bowel, and eye [1]. 

According to the estimations provided by the American Cancer Society (ACS) in 2010, 68.130 new cases of melanomas were diagnosed and approximately 8.700 people died from this cancer [2]. The incidence of melanoma in the US has increased of about three folds between the last three decades (i.e., from 7.89 per 100.000 in 1975 to 22.52 per 100.000 in 2008) [3]. Clinical and epidemiological data suggest that several risk factors can contribute to the increased incidence: (i) extensive or repeated exposure to sunlight [4]; (ii) individuals with family history of melanoma (5–12% of all reported cases) [5]; (iii) high nevi count and dysplastic nevus [6], thereby suggesting the need to perform a biopsy of the suspicious lesion. The biopsy permits to establish not only an accurate diagnosis but also to define the optimal staging and proceed earlier with the appropriate therapy (e.g., surgery, chemotherapy, and/or radiotherapy).

Metastatic melanoma (i.e., advanced malignant melanoma) is the most aggressive form of skin cancer with a median overall survival (OS) of only few months (8 to 18 months) [2]. This fact could be mainly explained by the modest results obtained with dacarbazine (DITC) and high-dose interleukin 2 (HD IL-2), the two unique FDA-approved therapies for metastatic melanoma until 2011 [7–9]. Indeed, DITC is limited by a low response rate (RR of 5% to 15%) and an insufficient OS (about 8 months) [7]. Besides, HD IL-2 is also limited by a low RR (6% to 10%), a short duration of responses in most patients as well as a severe toxicity [8, 9]. Since 2011, 3 new agents have been approved for the treatment of advanced melanoma by the Food and Drug Administration (FDA) [10–12]: (i) vemurafenib, a mutant *B*-*RAF* ^V600E^ inhibitor, recommended for unresectable or metastatic melanoma [10]; (ii) ipilimumab, an anti-CTLA-4 monoclonal antibody, also preconized for the treatment of unresectable or metastatic melanoma [11]; (iii) pegylated interferon alpha-2b (PEG-IFN), a covalent conjugate of the polyethylene glycol (PEG) with the recombinant *α*-2b interferon (IFN), long-time used to treat chronic hepatitis patients infected with hepatitis c virus [12], and currently recommended as adjuvant treatment for stage III melanoma [13].

Vemurafenib has emerged as a highly selective mutant *B*-*RAF* ^V600E^ inhibitor with little effect on wild type *B-RAF*, thereby demonstrating significant tumor regression (>40%) while minimizing side effects in a large number of patients with metastatic melanoma [14–16]. Nevertheless, the single use of this oncogene-targeted agent presents the following main disadvantages: (i) short median duration of response (MDR) and progression-free survival (PFS) (i.e., about 6 months only) [16]; (ii) low RR in patients who harbor mutations other than V_600_E. The proportion of these patients ranges between 10% and 30% (e.g., V_600_K is present in 5% to 20% of patients with melanoma) [17, 18].

Ipilimumab was designed and developed to block the cytotoxic T-lymphocyte-associated antigen 4 (CTLA-4), thereby increasing the T-cell activity and promoting antitumor activity in patients with cancers [19]. Thereby, in patients with unresectable or metastatic melanoma, ipilimumab plus DITC versus DITC alone significantly improved the OS (about 11 months versus 9 months, resp.) and RR (about 15% versus 10%, resp.) [20, 21].

PEG-IFN, similarly to high-dose interferon (HDI or Intron A) [1, 22], has been approved for the adjuvant treatment (after surgical resection) of stage III melanoma patients. This approval was mainly based on final results of a recent randomized phase III trial organized by the European Organization for Research and Treatment of Cancer (EORTC) 18991 that showed greater relapse-free survival (RFS of 45.6%) in comparison to observation (38.9%), although no significant effect on OS was noticed [12, 23]. Previously, in an open-label phase 2 study, the efficacy and safety of PEG-IFN in combination with temozolomide were investigated in patients with metastatic melanoma without brain metastases [24]. The RR of this combination reached 31% of the patients, the median OS was 12 months, and no patient developed brain metastases while receiving study treatment, which was besides well tolerated. Up to date, and in the best of my knowledge, it remains unknown whether PEG-IFN can provide better efficacy and safety results than the biochemotherapy combining cisplatin, vinblastine, DTIC plus IL-2 (Proleukin), and interferon. Indeed, a recent phase 3 trial (SWOG S0008) only assessed the efficacy and safety of this biochemotherapy versus HDI in patients with high-risk melanoma [25]. The results showed major improvement in the median RFS in favor of biochemotherapy (4.3 years versus 1.9 years with HDI). OS, however, was exactly the same (56% at 5 years), and acute grade 4 toxicity was more frequent with biochemotherapy. All together, the biochemotherapy can be considered as a better adjuvant treatment than HDI. Currently, both HDI and PEG-IFN are considered as category 2B due to their limited benefits, and so will not be further detailed in this paper.

Eventually, in one hand, these new exciting chemotherapies represent a great hope for the physicians and patients with advanced melanoma. In the other hand, their respective limitations clearly emphasize the importance of developing novel treatment strategies (e.g., cell-based therapies, advanced and rational combinatorial therapeutic approaches, nanodrug formulations). These alternative therapeutic options might help to improve OS, PFS, RFS, RR, and MDR while minimizing toxic adverse events, thereby contributing *in fine* to the quality of the patient's life.

In this paper, most recent FDA-approved treatments against advanced cutaneous melanoma (stage III and IV) are highlighted, and possible enhanced therapeutic strategies to overcome their respective associated limitations are discussed.

## 2. Latest FDA-Approved Drugs for Advanced/Metastatic Melanoma

Most patients with unresectable stage III or stage IV disease require systemic treatment rather than metastasectomy.

### 2.1. Oncogene-Directed Therapy: Mutant B-RAF Inhibitors

Melanoma is a molecularly heterogeneous disease with approximately half (40%–60%) of the cutaneous melanoma cells harboring an activating mutation in the *B-RAF* gene, which encodes a serine/threonine kinase protein kinase, and most of the mutations (>70%) are V_600_E (i.e., substitution of valine for glutamate at amino acid position 600) [17, 26–28]. Because mutated *B-RAF* leads to constitutive activation of the mitogen-activated protein kinase pathway (MAPK) that, in turn, increases the cellular proliferation and drives the oncogenic activity [29, 30], intensive research has consisted to selectively inhibit mutated *B-RAF* in patients with melanoma (e.g., studies with sorafenib, a multitargeted kinase inhibitor), but the results were globally disappointing due to off-target side effects mainly induced through inhibition of wild type *B-RAF* [14, 31–35].

Among highly selective *B-RAF* inhibitors, only the recent FDA-approved vemurafenib (formerly PLX4032, currently marketed as Zelboraf and initially developed by Genentech Roche) is capable of silencing mutant *B*-*RAF* ^V600E^ without interfering with wild type *B-RAF*. Indeed, in a phase 2 clinical trial involving patients with metastatic melanoma harboring *B*-*RAF* ^V600E^ mutation (*n* = 132), vemurafenib demonstrated substantial tumor regression in 81% of the cases, a RR of 52%, and a MDR of 6.8 months [14–16]. Further, in a phase 3 clinical trial (BRIM3) involving previously untreated patients (*n* = 675), vemurafenib was much better than DITC in terms of RR (48% versus 5%, resp.), PFS (5.3 months versus 1.6 months, resp.), and percent of patients alive at six months (OS of 84% versus 64%, resp.) [10]. Also, in a recent open-label pilot study, it was stated that vemurafenib could be beneficial for previously treated metastatic melanoma patients with brain metastases [36]. Besides, common adverse events associated with vemurafenib included accelerated growth of cutaneous squamous cell carcinomas (SCCs) and keratoacanthomas [10, 37–40], most probably through paradoxical activation of MAPK signaling (about 20–25% of the patients with advanced melanoma) [37–40]. 

Eventually, vemurafenib represents an excellent model for successful targeted anticancer therapy (i.e., high RR and low toxicity) in patients with *B*-*RAF* ^V600E^ mutations [41]. Nevertheless, these clinical benefits are counterbalanced by the relatively short MDR, high selectivity for the *B*-*RAF* ^V600E^ mutation, and related toxicities of the drug. Owing to consideration that 10% to 30% of patients have a non-*B* *RAF* ^V600E^ mutation (e.g., *B*-*RAF* ^V600k^ mutation is present in 5% to 20% of melanoma patients) [17, 18], further studies are required to examine the efficacy of vemurafenib, alone or in combination, in patients with a non-*B* *RAF* ^V600E^ mutation. These studies are important to avoid useless administration of vemurafenib in a subset of patients, who might otherwise become resistant to the drug. Alternatively, rational combination of vemurafenib with other agents (e.g., ipilimumab) might circumvent an eventual drug resistance and/or further improve the clinical outcome of the patients (e.g., MDR, OS). 

### 2.2. Immunotherapy: CTLA-4 Inhibitors

Melanoma is one of the most immunogenic tumors due to the presence of tumor infiltrating lymphocytes (TIL) in resected melanoma, clinical responses to immune stimulation, and occasional spontaneous regressions. CTLA-4 expression is necessary for activation of self-regulation of T cells, and so CTLA-4 inhibitors could represent serious therapeutic options to generate T-cell hyperresponsiveness and overcome tumor immune escape [19].

Up to date, ipilimumab (formerly MDX-010, MDX-101, or MDX-CTLA-4, currently marketed as Yervoy and initially developed by Bristol-Myers Squibb) is a fully human IgG1 monoclonal antibody that blocks CTLA-4, subsequently increasing the T-cell activity and promoting an antitumor activity and represents the only approved immunotherapeutic agent for systemic treatment [20]. In the first phase 3 randomized trial involving patients with previously treated unresectable stage III or IV melanoma (*n* = 676), ipilimumab compared to the glycoprotein 100 peptide (gp100) vaccine demonstrated an improved median OS (10.1 months versus 6.4 months, resp.) and a much better RR (10.9% versus 1.5%, resp.), *albeit* the occurrence of toxicities with ipilimumab, including grade 3 or 4 immune-related adverse events (e.g., enterocolitis, hepatitis, and dermatitis) and deaths, was higher than with gp100 (10–15% versus 3%, resp.) [11]. Ipilimumab plus gp100, compared to gp100, did not improve the OS observed with ipilimumab alone (10.0 months versus 10.1 months, resp.) [11]. In the second phase 3 randomized trial involving previously untreated patients with metastatic melanoma (*n* = 502), ipilimumab combined with DITC demonstrated a modest but statistically significant improvement in OS compared to DITC plus placebo (11.2 months versus 9.1 months, resp.) as well as a better overall RR (15.2% versus 10.3%, resp.) [20, 21]. Interestingly, survival rates over the years were always significantly higher in the ipilimumab-DITC group than in the group treated with the single agent DITC (at 1 year: 47.3% versus 36.3%; at 2 years: 28.5% versus 17.9%; at 3 years: 20.8% versus 12.2%, resp.), clearly demonstrating that ipilimumab is able to confer a durable response (MDR of 19.3 months versus 8.1 months, resp.). Nevertheless, median PFS was barely improved (2.8 months versus 2.6 months, resp.). Also, grade 3 or 4 adverse events (e.g., hepatitis) occurred more frequently in patients treated with ipilimumab plus DITC than in patients treated with DITC (plus placebo) (56.3% versus 27.5%, resp.), although low rates of gastrointestinal events and no drug-related deaths occurred in the ipilimumab-DITC group [20, 21].

Eventually, although the OS and MDR noticed with ipilimumab are higher than that one observed with vemurafenib, the most important limitation of this drug tested alone or in combination remains the modest RR. This strongly suggests a need for rational combination between ipilimumab and other commercially available free- or nanoencapsulated drugs (e.g., vemurafenib and bevacizumab, resp.) that might provide complementary clinical benefits. 

## 3. Therapeutic Perspectives for Improving the Effects of FDA-Approved Drugs

### 3.1. Promising Molecular Targets to Overcome the Resistance Associated with B-RAF Inhibitors

Since approximately 50% of patients with melanoma harbor *B-RAF *mutations and might be then eligible for treatment with the novel *B-RAF* inhibitors, this means that another half of the patients with advanced melanoma might not fully benefit from vemurafenib (i.e., specific *B*-*RAF* ^V600E^ targeting drug). Therefore, it appears reasonable to develop drugs that specifically target gene mutations other than V_600_E (e.g., *B*-*RAF* ^V600k^ targeting agents). Also, at a larger extent, targeting any molecular alterations that occur (frequently or rarely) in specific melanoma related pathways (including those which are involved in “melanoma stem cells”) shall provide additive or synergistic clinical benefits. For instance, molecules involved in the mitogen-activated protein kinase (MAPK) signaling could represent interesting targets to overcome the melanoma resistance. Indeed, it has been previously shown that MAPK activation by mutants *B-RAF* drives melanoma tumor proliferation, and that the resistance to *B-RAF* inhibitors—responsible for their short-duration response—can be (or not) associated with reactivation of the MAPK pathway (“escape route”) [41–43]. Recent studies demonstrated that MAPK-dependent acquired resistance to *B-RAF* inhibition in melanomas can involve (i) upregulation of receptor tyrosine kinases (RTKs) and N-RAS [44]; (ii) elevated expression of COT kinase through MAPK pathway reactivation [45]; (iii) activation of MAPK kinase (MEK *aka* MAPK/ERK) [46, 47]; (iv) elevation of C-RAF [48]. Besides, MAPK-independent molecules involved in the melanoma resistance mechanism include (i) upregulation of IGF-1R [49]; (ii) PI3K/AKT signaling through the activation of c-KIT (a RTK also known as CD117) [50]; (iii) loss of PTEN through the suppression of BIM expression [51]. 

Recent promising results from clinical trials mainly implicate MEK-1 and c-KIT inhibitors. Indeed, a recent phase III trial (METRIC study) involving patients with advanced or metastatic melanoma harboring *B*-*RAF* ^V600E/k^ mutant and without prior brain metastases (*n* = 332) reported the great efficacy of a reversible and highly selective allosteric inhibitor of MEK1/2 activity, trametinib (formerly GSK 1120212), when compared with “chemotherapy” (DITC or paclitaxel, but not vemurafenib) [52]. Thereby, significant improvements have been observed in favor of trametinib, mainly at the median PFS (4.8 months versus 1.4 months with chemotherapy) and the overall RR (24% versus 7% with chemotherapy). In spite of crossovers, at 6 months, OS was also significantly improved in favor of trametinib (74% versus 56% with “chemotherapy”). Globally, trametinib was well tolerated and safe (e.g., most side effects included skin rash and hypertension), validating that targeting the MEK pathway is a viable strategy. Besides, ongoing trials are evaluating the safety and efficacy of c-KIT inhibitors (i.e., imatinib, nilotinib, and dasatinib) [53–55], and their combinations with *B-RAF* inhibitors are underway. Indeed, *c-KIT * is mutated in approximately 20% of sun-damaged skin [56] and, once its corresponding protein is activated by its ligand called SCF (Stem Cell Factor), mediates cell growth and cell survival signals through signaling pathways such as P13K-AKT-mTOR and RAS-RAF-MEK-ERK. Thereby, *c-KIT* has been implicated in the pathogenesis of several cancers including metastatic melanoma [57, 58], and previous clinical trials demonstrated a durable overall RR (16–24%) as well as a median OS ranging from 11 to 14 months [50, 53, 59].

Eventually, rational combination of *B-RAF* inhibitors (e.g., vemurafenib, dabrafenib) with drugs that specifically target one of the above-mentioned molecules (e.g., MEK, c-KIT, or CTLA-4) would further improve the clinical outcome of the patients with advanced/metastatic melanoma. A current nonrandomized open-label phase 1/2 trial (NCT01400451 study) that aims to evaluate the efficacy and safety of vemurafenib with ipilimumab in adult subjects with *B*-*RAF* ^V600^ mutation-positive metastatic melanoma is ongoing but is not recruiting participants, yet [60]. The results of this challenging study are expected for August 2015 [60]. Interestingly, the selective *B-RAF *inhibitor, dabrafenib (formerly GSK2118436), previously showed similar efficacy to vemurafenib [35], and several clinical trials in patients with *B*-*RAF* ^V600^ mutantmelanoma showed promising results with dabrafenib as a single agent [61–63] or in combo with trametinib [64–66]. Thereby, a recent multicentre, open-label phase 1/2 trial (BREAK-MB study), investigated the efficacy and safety of oral dabrafenib in adult patients with *B*-*RAF* ^V600E/k^ mutant melanoma metastatic to the brain (*n* = 172) [61, 62]. 29 of 74 (39.2%) patients with *B*-*RAF* ^V600E^ mutantmelanomaand only 1 of 15 (6.7%) patients with *B*-*RAF* ^V600k^ mutant melanomain cohort A (*n* = 89 patients who had not received previous local treatment for brain metastases) achieved an overall intracranial response with dabrafenib. Comparatively, 20 of 65 (30.8%) patients with *B*-*RAF* ^V600k^ mutant melanoma and 4 of 18 (22%) with *B*-*RAF* ^V600k^ mutant melanomain cohort B (*n* = 83 patients who had progressive brain metastases after previous local treatments) achieved an overall intracranial response with dabrafenib. Further, the treatment-related adverse events of grade 3 or worse occurred in 38 (22%) patients from the cohort A and in 51 (30%) patients from the cohort B. The three most frequent serious events were pyrexia (6%), intracranial hemorrhage (6%), and SCC (6%). To sum up, these impressive results were mainly obtained in patients with *B*-*RAF* ^V600E^ mutant melanoma and brain metastases, irrespective of whether they were previously treated or untreated, clearly showing that dabrafenib is safe (e.g., lack ofskin toxicity) and can confer robust activity in intracranial disease, suggesting possible elimination of whole brain radiotherapy in similar subsets of patients. Further, in a randomized open-label multicenter phase 3 trial (BREAK-3 study) involving previously untreated patients with *B*-*RAF* ^V600E^ mutant melanoma (*n* = 250) [63], dabrafenib compared to DITC led to (i) a significant improvement of the median PFS (5.1 months versus 2.7 months, resp.); (ii) a greater RR (53% versus 19%, resp.). The OS data were immature at the time of the study analysis. Further, hyperkeratosis (37%), pyrexia (28%), and skin papillomas (24%) were among the most frequent adverse events observed in patients treated with dabrafenib, and interestingly, only few cases of SCC (7%) and keratoacanthomas (3%) were noticed [63]. Other recent results, obtained from an open-label study involving patients with metastatic melanoma and *B*-*RAF* ^V600^ mutations (*n* = 247), showed that dabrafenib and trametinib can be safely combined at full monotherapy doses (150 mg and 2 mg, resp.) [64–66]. Indeed, for the combo therapy group of patients, compared to the monotherapy group (dabrafenib only), the following end points were observed (i) the rate of pyrexia was increased (71% versus 26%, resp.); (ii) the rate of proliferative skin lesions (i.e., the incidence of cutaneous SCC) was not significantly reduced (7% versus 19%, resp.); according to me, this observation might be explained by the presence of MEK-independent subsets of mutant RAS, since MEK-dependent and -independent subsets of mutant RAS have been identified in tumor cell lines [67, 68]; (iii) PFS was significantly improved (9.4 months versus 5.8 months, resp.); (iv) RR was significantly improved (76% versus 54%, resp.); (v) the duration of response was also much better (10.5 months versus 5.6 months, resp.). However, OS could not be compared because of the relatively short-term followup. These overall exciting experimental results will need to be confirmed in ongoing phase 3 larger studies before combo therapy likely replaces monotherapy (i.e., *B-RAF* inhibitor as a single agent). Indeed, larger studies might demonstrate whether *B-RAF*/MEK combo therapy can (i) both delay and prevent onset of resistance to therapy; (ii) significantly reduce the incidence of SCC formation fevers, chills, and MEK inhibitor-induced dermatitis; (iii) extend overall survival or if ipilimumab shall be implemented to confer a survival benefit in case of treatment failures (e.g., disease progression, unacceptable adverse events); (iv) recover patients that initially failed with monotherapy or provide potential benefit to initial monotherapy in order to further improve PFS.

### 3.2. Promising Molecular Targets to Overcome Low-Response Rate Associated with Immunotherapeutic Agents

Although ipilimumab can induce long-term responses in a subset of patients, the relatively low RR (10%–15%) observed with this immunotherapeutic agent limits its use. Possible ways to overcome this limitation would be (i) increase of the dose (e.g., 3 to 10 mg/kg); (ii) selection/stratification of the patients (i.e., personalized medicine); (iii) rationale combination with other treatment modalities (e.g., molecularly targeted therapy, radiation therapy, adoptive T-cell therapy, melanoma initiating/propagating cells).

The low response rate of ipilimumab is thought to be caused by melanoma immune escape, a mechanism that involves the expression of programmed death ligand 1 (PD-L1), which once bound to its ligand PD-1 (programmed death-1) of activated lymphocytes, would cause apoptosis of the activated lymphocytes and subsequent immune tolerance [69–71]. Interestingly, a phase 1 study led on specific human anti-PD-1 antibody (formerly MDX-1106 *aka* BMS-936558), a fully recombinant human immunoglobulin G4 [72], offered clinical benefits in a variety of previously treated refractory solid tumors including melanoma, without generating significant toxicities [72]. Thereby, a dose-escalation study of the combination of MDX-1106 with ipilimumab has been started [53], and phase 1 trials of anti-PD-L1 are also underway. Recently, the clinical activity and safety of MDX-1106 intravenously administrated in escalating doses every two weeks in patients with advanced melanoma (*n* = 94), have been reported [73]. The overall RR was 28%, and 6% of patients achieved stable disease after24 weeks. Most side effects were immune related, though three treatment-related deaths occurred on study, including two due to pneumonitis. Interestingly, a subanalysis of the data hinted that the PD-L1 protein might serve as a biomarker of response. Indeed, more than one-third of patients expressing PD-L1 responded to immunotherapy, whereas responses were not observed among patients lacking PD-L1 expression. Eventually, these preliminary data strongly suggest that blockage of the PD-1 pathway may represent a new immune therapy.

Besides, pertinent clinical studies are assessing the combinatorial effects of ipilimumab with promising antiangiogenic drugs (e.g., bevacizumab, an antivascular endothelial growth factor A (VEGF-A)), immunostimulators (e.g., sargramostim, a recombinant human granulocyte macrophage colony-stimulating factor (GM-CSF)), and/or anticancer agents (e.g., fotemustine, a nitrosourea alkylating agent, or the *B-RAF* inhibitor vemurafenib) based on previous studies [74–76]. Indeed, a recent randomized phase 2 trial (BEAM study) involving patients with previously untreated advanced melanoma (*n* = 214) has evaluated the activity of the FDA-approved drug bevacizumab (Avastin) combined with carboplatin plus paclitaxel (BCP) versus placebo carboplatin plus paclitaxel (CP) [74]. Although the results were not as good as expected (median PFS: 5.6 months with BCP versus 4.2 months with CP; overall RR: 25.5% with BCP versus 16.4% with CP; median OS: 12.3 months with BCP versus 8.6 months with CP), the trial might be considered as positive. Further, no new safety signals were observed. Interestingly, the hazard rate (0.79) for improvement in OS at the last time point was the same seen with BCP that led the US FDA to approve bevacizumab for lung cancer [75]. Therefore, a trial of chemotherapy combining bevacizumab with the first-line therapeutic agents for metastatic melanoma (e.g., ipilimumab, vemurafenib) might be beneficial. In this regard, the first combination study, a recent nonrandomized open-label phase 1 trial, did investigate potential synergies of ipilimumab and bevacizumab in a limited number of evaluable adult patients with unresectable stage III or stage IV melanoma (*n* = 21) [76]. This preliminary and promising study showed that ipilimumab plus bevacizumab could (i) display synergistic effects and provide clinical benefit in a large number of patients as shown in 14/21 of the enrolled patients; (ii) be safely administered with management of noted toxicities, which were mostly immune related (e.g., grade 3-4 hepatitis, grade 2 colitis, arteritis, hypophysitis, thyroiditis, bilateral uveitis,). Further, studies with the immunostimulator sargramostim (Leukine/Prokine) show that this drug may help the immune system recover from the side effects of treatment in patients with metastatic melanoma and so could be tested as a combinatory agent with ipilimumab. Indeed, a previous investigation (NCCTG study) has consisted to perform a dose-escalation clinical trial with aerosolized sargramostim in HLA-A2 patients with metastatic melanoma to the lung (*n* = 40). The data showed that the toxicity was acceptable for all tested doses and the greatest increase in antitumor T-cell immune responses was achieved at the highest doses [77]. Then, a hypothesis-generating study was conducted to evaluate the safety and efficacy of prolonged administration of sargramostim as surgical adjuvant therapy in patients with melanoma at high risk of recurrence (*n* = 98) [78]. The prolonged administration of the drug was well tolerated among patients, and the 5-year melanoma-specific survival rate was 60%, providing preliminary evidence that sargramostim can be administered as adjuvant therapy for patients with melanoma at high risk of recurrence. Interestingly, an ongoing recent randomized open-label phase 2 longitudinal trial (NCT01134614) is studying how well giving ipilimumab with (arm I) or without (arm II) sargramostim works in treating adult patients with stage III or stage IV melanoma without brain metastases (estimated enrollment *n* = 220) [79]. However, the effects (e.g., OS, PFS, RR, MDR, safety) of ipilimumab plus sargramostim versus ipilimumab alone, in treating patients with advanced melanoma, remain unknown. Eventually, fotemustine (Muphoran), a non-FDA-approved drug available in Europe, is known to rapidly cross the blood-brain barrier and to display encouraging activity in patients with brain metastases [80]. Also, in a phase 3 trial involving patients with metastatic melanoma (*n* = 229) [81], fotemustine compared to DITC was associated with (i) an improved overall RR (15.5% versus 6.8%, resp.); (ii) a trend toward improved OS (7.3 versus 5.6 months, resp.); (iii) a longer median time to development of brain metastases (22.7 months versus 7.2 months, resp.) in patients without brain metastases at inclusion; (iv) a similar MDR (5.8 months versus 6.9 months); (v) a similar time to progression (1.8 months versus 1.9 months); (vi) a similar quality of life; (vii) more adverse events such as myelosuppression (i.e., grade 3 to 4 neutropenia 51% versus 5%, resp. and thrombocytopenia 43% versus 6%, resp.) and alopecia. Interestingly, a recent open-label, single-arm phase 2 trial (NIBIT-M1 study) is investigating the efficacy and safety of ipilimumab plus fotemustine in adult patients (*n* = 86) with metastatic melanoma and with or without asymptomatic brain metastases [82]. As primarily results, this medicinal combination achieved disease control in 40 patients (46.5%) with metastatic melanoma, including those with brain metastases. Nevertheless, the treatment-related adverse effects (e.g., grade 3 or 4 myelotoxicity and hepatotoxicity) were present in 47 patients (55%).

Eventually, the use of nanomaterials to formulate nanodrugs (e.g., drug nanocarriers such as solid lipid nanoparticles (SLN) or nanostructured lipid carriers (NLC)) might be useful to possibly enhance efficacy (e.g., systemic bioavailability, targeting), tolerability, and safety of free drugs presenting clinical benefit for patients with metastatic melanoma (e.g., ipilimumab, vemurafenib). In general, it is well accepted that administration of appropriate nanodrug formulations can contribute to enhance the duration response, the response rate, the OS, and PFS in patients with a given type of cancer [83, 84]. In this regard and based on a recent study announcing the preparation of nanoliposomes-encapsulated bevacizumab with beneficial effects in prolonging the residency of bevacizumab in the vitreous [85], it might be interesting to test ipilimumab plus nanoencapsulated bevacizumab in patients with unresectable stage III or stage IV melanoma. Besides, “adoptive T-cell therapy” (ACT), which presents several conceptual similarities with hematopoietic stem-cell transplantations (HSCTs) in terms of advantages and disadvantages can constitute another therapeutic option to overcome the low response associated with immunotherapeutic agents (e.g., ipilimumab). Indeed, successful isolation-expansion-infusion of TIL has shown clinical benefits for the treatment of patients with metastatic melanoma [86–91]. For instance, the RR (72%) was higher when using nonmyeloablative lymphodepletion with cytotoxic chemotherapy and with or without total body irradiation (TBI) than when ipilimumab was combined with IL-2 [86–91]. Interestingly, preclinical work suggested that selective *B*-*RAF* ^V600E^ inhibition enhances T-cell recognition of melanoma without affecting lymphocyte function, providing a rational for the combination of *B-RAF* inhibitors (e.g., vemurafenib or dabrafenib) with stimulatory immune agents (e.g., ipilimumab or PD-1/PD-L1) [92].

## 4. Conclusions

The incidence of metastastic melanoma is increasing worldwide. Vemurafenib and ipilimumab, based on their respective success rates, are bringing hopes to physicians and patients. Vemurafenib has emerged as a highly selective *B*-*RAF* ^V600E^ mutant melanoma inhibitor and could display good response rates in patients with unresectable or metastatic melanoma. Nevertheless, its main disadvantage remains the short median duration response as well as its limited use to patients who harbor mutations other than *B*-*RAF* ^V600E^ (e.g., *B*-*RAF* ^V600k^). Besides, ipilimumab was developed to block the cytotoxic T-lymphocyte-associated antigen 4 (CTLA-4) in patients with unresectable or metastatic melanoma. Interestingly, ipilimumab presents the opposite main advantage and disadvantage than vemurafenib. Several clinical trials are underway to address the question of rational combination of those two approved drugs, together and/or separately with other potential therapeutic targets (PD-1, PD-L1, c-KIT, MEK1, VEGF-A…). Cell therapy such as adoptive T cell is encouraging. Nanoencapsulation of the recent FDA-approved free drugs (e.g., vemurafenib, ipilimumab), using proper nanocarriers, or rational combination of these free drugs with available adjuvant nanotherapeutics might be beneficial as they might enhance the overall pharmacological features (e.g., bioavailability and targeting). The importance of direct targeting of potential melanoma initiating/propagating cells within a given patient was not detailed in this paper but might be important in order to avoid immune resistance, immune escape, and disease relapse. Owing to consideration that the incidence of advanced/metastatic melanoma in the younger population is increasing, clinical trials in pediatric patients appear necessary. Eventually, the rational molecular or cellular combo therapy is a key strategy, as it shall beneficiate a larger number of patients. The recent results reported in this paper are quite exciting and, undeniably, constitute a new hope for patients and healthcare professionals.

## Abbreviations

ACT: Adoptive T-cell therapy
ACS: American cancer society
BCP: Bevacizumab/carboplatin/paclitaxel
CTL-A4: Cytotoxic T-lymphocyte-associated antigen 4
DITC: Dacarbazine
FDA: Food and drug administration
HDI: High-dose interferon
HSCT: Hematopoietic stem cell transplantation
IL-2: Interleukin-2
PD-1: Programmed death-1
PEG-IFN: Pegylated interferon alpha-2b
MAPK: Mitogen-activated protein kinase
MDR: Median duration response
MEK: MAPK/extracellular signal-regulated kinase (ERK)
MEKi: MEK inhibitor
OS: Overall survival
PEG: Polyethylene glycol
PFS: Progression-free survival
RAF: Ras-activating factor
RFS: Relapse/recurrence free survival
RR: Response rate
SCC: Squamous cell carcinoma
TBI: Total body irradiation
TIL: Tumor infiltrating lymphocytes.
